# Fidaxomicin’s Role in Overcoming Vancomycin Failure in Clostridium difficile Infections: A Case Series and Literature Review

**DOI:** 10.7759/cureus.81110

**Published:** 2025-03-24

**Authors:** Mohammad Kloub, Shefali Pati, Ahmad W Haddad, Yazeed Abu Ruman, Qusai Al-Maharmeh, Mohammad Nabil Rayad, Bereket Tewoldemedhin, Jihad Slim

**Affiliations:** 1 Department of Internal Medicine, Saint Michael's Medical Center, Newark, USA; 2 Department of Internal Medicine, St George's University, True Blue, GRD; 3 Department of Gastroenterology and Hepatology, Saint Michael's Medical Center, Newark, USA; 4 Department of Infectious Diseases, Saint Michael's Medical Center, Newark, USA

**Keywords:** clostridium difficile, clostridium difficile infection, clostridium difficile infection treatment, diarrhea, fidaxomicin, vancomycin

## Abstract

*Clostridium difficile* infection (CDI), characterized by diarrheal illness with serious complications, is a common pathology in clinical practice. We present a series of five patients with CDI who underwent treatment with fidaxomicin following the failure of oral vancomycin. To our knowledge, no evidence in the literature suggests that fidaxomicin is more effective than vancomycin in treating acute infection. This paper emphasizes the importance of utilizing a large study to determine the relative effectiveness of vancomycin versus fidaxomicin in treating CDI. We also provide a literature review on CDI and management evolution.

## Introduction

*Clostridium difficile*, also called *C. difficile*, is a gram-positive spore-forming obligate anaerobic bacteria that frequently inhabits the large intestine of humans. Colonization with *C. difficile* is not hazardous since its growth is inhibited by other bacteria in the digestive tract. However, *C. difficile* can develop in its vegetative state and produce toxins that harm the intestinal epithelium in specific situations, such as after gastrointestinal surgery or antibiotics [1]. *Clostridium difficile* infection (CDI) can result in a high temperature, diminished appetite, and gastrointestinal symptoms, including diarrhea, nausea, and abdominal pain. Complications from severe CDI might include sepsis, pseudomembranous colitis, and even death [2,3].

The number of deaths linked to CDI is increasing, and the illness is striking groups traditionally thought to be at low risk, including young and physically fit community members [4]. The lower rates of clinical response and higher rates of recurrence observed in more recent trials are concerning compared to data from the mid-1990s [5].

CDI treatment guidelines have been changing with time. In recent years, vancomycin or fidaxomicin have replaced metronidazole and vancomycin as the pillars of CDI therapy [6]. We present a series of patients with CDI who have shown significant clinical improvement with fidaxomicin after failing treatment with vancomycin. This paper aims to highlight the advantages of fidaxomicin over vancomycin as a treatment for CDI; we also provide a comprehensive review of *C. difficile* pathogenesis and treatment options over time.

## Case presentation

Case 1

A 56-year-old male with a medical history including hypertension, diabetes mellitus, antiphospholipid syndrome, deep vein thrombosis, atrial fibrillation, multiple strokes, and a previous episode of CDI nine months prior to admission was treated with intravenous metronidazole and oral vancomycin. The patient arrived at the emergency department (ED) complaining of four days of intermittent lower abdominal pain. He reported experiencing watery diarrhea seven to nine times per day, with two episodes of nausea and vomiting; no blood was observed in vomitus or diarrhea. Two weeks before admission, he had been prescribed Augmentin and steroids for upper respiratory tract symptoms. Upon admission, the patient was afebrile and vitally stable. Upon examination, he appeared dehydrated, and the abdominal examination was unremarkable.

**Figure 1:**
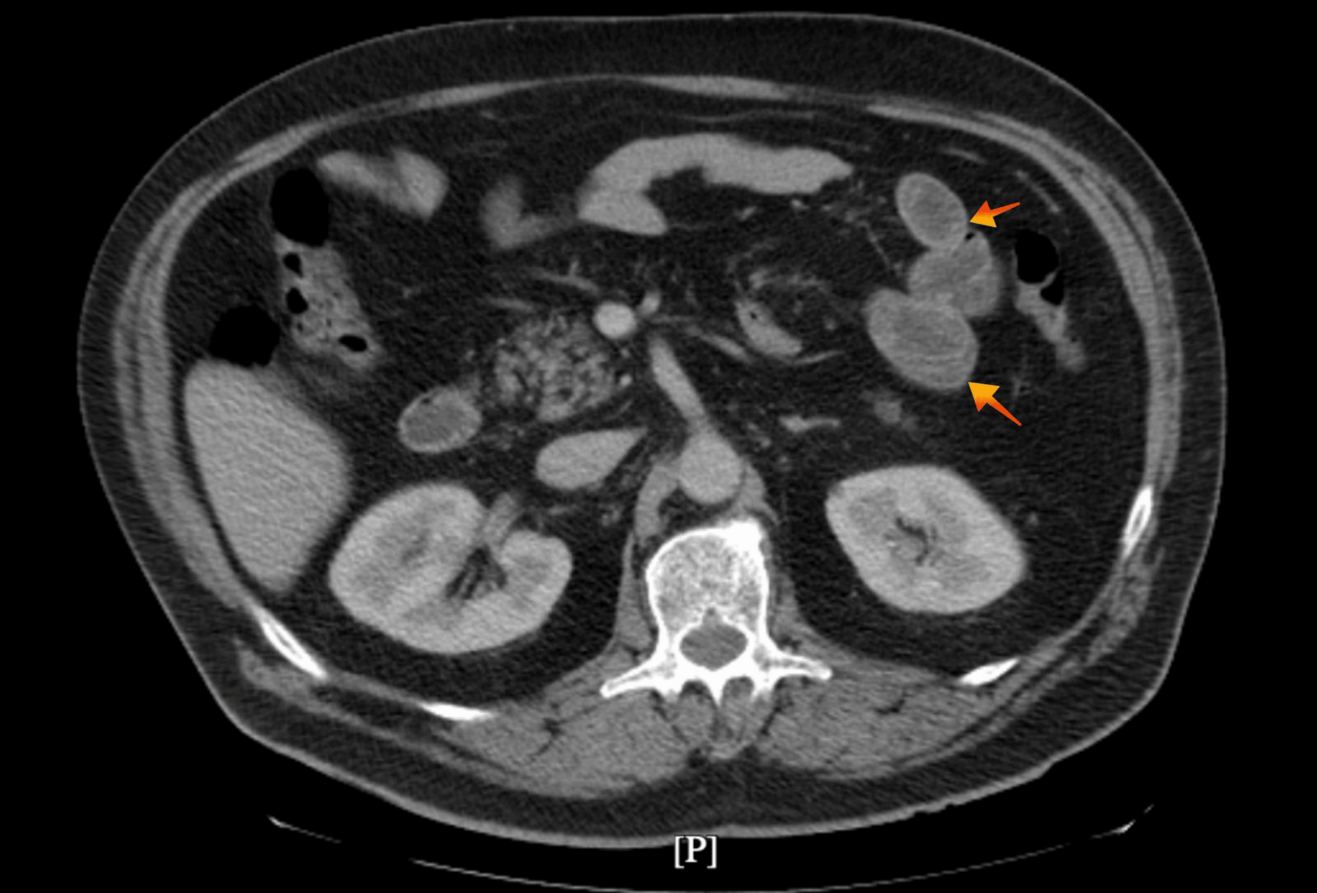
Computed tomography (CT) scan revealed features of acute enteritis. Bowel wall thickening and mucosal enhancement (arrows).

The *C. difficile* polymerase chain reaction (PCR) and the toxin test were positive, and a computed tomography (CT) scan revealed features of acute enteritis (Figure 1). Initial labs showed a white blood cell count (WBC) of 13x10^3 cells/μL (normal range 4.0-10.0x10^3 cells/μL) and a normal creatinine level; other labs were within normal limits. He initially received oral vancomycin (125 mg every six hours) for four days, but his symptoms were worsened with 15 episodes of watery diarrhea and a WBC of 14x10^3 cells/μL (normal range 4.0-10.0x10^3 cells/μL). The treatment was switched to fidaxomicin 200 mg twice a day, leading to the resolution of diarrhea and normalization of WBC to 8x10^3 cells/μL (normal range 4.0-10.0x10^3 cells/μL). The patient was discharged with fidaxomicin and had an outpatient follow-up with no recurrence of symptoms.

Case 2

A 63-year-old male with no previous medical history was brought to the ED after his family found him lying on the bed, soiled with urine and feces. He reported experiencing watery diarrhea mixed with soft stool, occurring five to six times a day for two to three weeks prior to admission. The patient denied any recent history of hospitalization or antibiotic use. On examination, the patient's blood pressure was 68/41 mmHg, afebrile, and heart rate was 89 beats per minute (BPM). He was alert and oriented to person, place, and time but displayed signs of dehydration. Abdominal examination revealed a soft abdomen without tenderness. 

**Figure 2:**
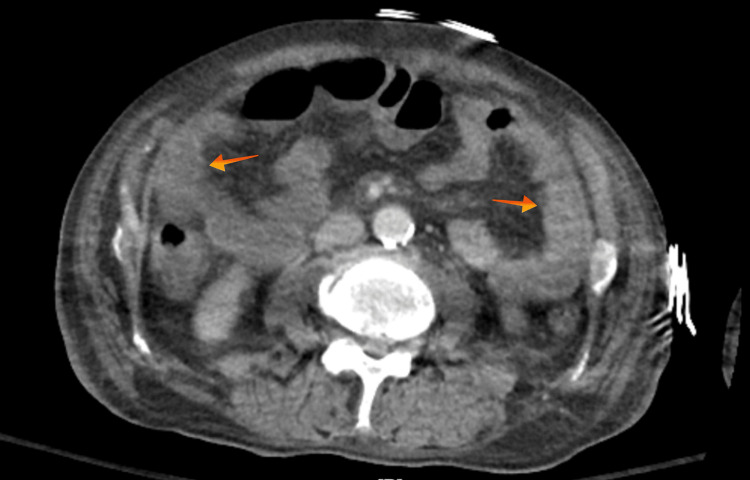
CT of the abdomen and pelvis with IV contrast revealed features of diffuse colitis involving the ascending, descending, and sigmoid colon (arrows).

Laboratory results showed a white blood cell count of 16x10^3 cells/μL (normal range 4.0-10.0x10^3 cells/μL), a creatinine level of 1.8 mg/dL (normal range 0.5-1.0 mg/dL), and a sodium level of 121 mEq/L (normal range 135-145 mEq/L). A CT of the abdomen and pelvis with IV contrast revealed features of colitis involving the ascending, descending, and sigmoid colon (Figure 2). The *C. difficile* PCR and the toxin test returned positive, while stool culture and ova and parasite tests were negative. The patient was initially admitted to the ICU for hydration and electrolyte replacement. A colonoscopy during admission revealed mild colitis in the cecum.

Despite receiving six days of oral vancomycin (125 mg every six hours) with IV metronidazole, the patient's symptoms initially remained the same and then started to worsen. Subsequently, vancomycin was switched to fidaxomicin 200 mg twice a day. A few days later, his symptoms improved, leading to the full resolution of diarrhea and normalization of the white blood cell count. 

Case 3

An 80-year-old female with a past medical history of diabetes, hypertension, hyperlipidemia, chronic kidney disease (CKD), and diverticulosis presented to the ED for lower GI bleeding and required subtotal colectomy as she was unstable. The patient was given piperacillin-tazobactam and metronidazole on the day of the surgery, and then she was off antibiotics afterward. Two weeks later, the patient started to have severe, watery, non-bloody bowel movements eight to 10 per day, and she spiked a high-grade fever, with normal blood pressure and pulse rate.

Her WBC was 14x10^3 cells/μL (normal range 4.0-10.0x10^3 cells/μL); her C-reactive protein (CRP) was elevated at 22.2 mg/dL (normal range 0.00-0.8 mg/dL); blood cultures showed no growth at five days. The *C. difficile* PCR and the toxin test returned positive. A CT scan with oral and IV contrast showed subtotal colonic resection with areas of induration in the peritoneum and retroperitoneum, presumably postsurgical.

Oral vancomycin 125 mg every six hours was started. CRP and WBC were trending down, but the patient kept complaining of severe watery diarrhea; on day five of vancomycin, metronidazole IV was added 500 mg twice a day; after eight days of vancomycin and three days of IV metronidazole, the patient's CRP was trending down, and her WBC was back to normal. However, the diarrhea did not improve, with seven to eight episodes every day, so the antibiotics were changed to fidaxomicin 200 mg twice a day; she completed ten days of treatment, and her watery diarrhea resolved entirely.

Case 4

A 42-year-old male with a past medical history of ulcerative colitis, deep vein thrombosis (DVT), and pulmonary embolism (PE) came to the ED complaining of abdominal pain. The pain started one month prior to admission when he had his screening colonoscopy. He came to the ED as the pain was progressive and accompanied by severe watery bowel movements, six to seven episodes every day; he denied any nausea or vomiting episodes. Upon physical examination, the patient was afebrile, and his blood pressure was 108/58 mmHg, with average pulse rate and oxygen saturation. Abdominal tenderness was found on deep palpation of the left lower abdomen, with no guarding or rebound tenderness. 

**Figure 3:**
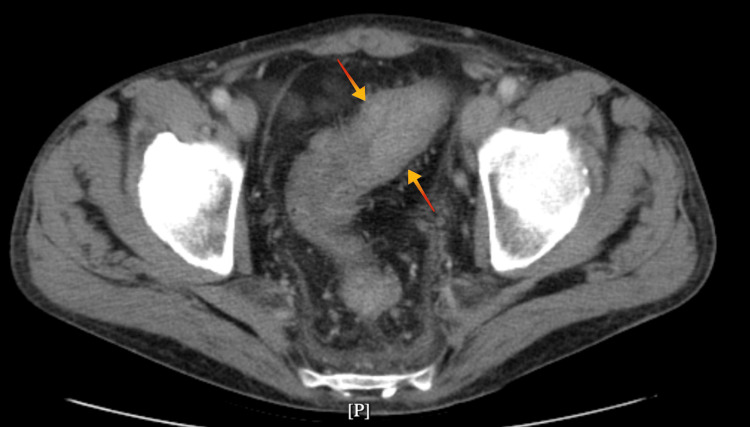
CT scan of the abdomen showing edematous and thickened bowel wall with contrast enhancement (arrows).

A CT scan showed edematous and thickened ascending, transverse, and descending colonic walls with acute inflammatory colitis (Figure 3). His CBC showed normal WBC, 9.1x10^3 cells/μL (normal range 4.0-10.0x10^3 cells/μL), and CRP was 10.3 mg/dL (normal range 0.00-0.8 mg/dL). *C. difficile* PCR and the toxin test were positive. The patient was started on oral vancomycin (125 mg every six hours) and IV metronidazole. For the first four days, his CRP was going back to normal. However, his bowel movement frequency was not improving, with an average of six to eight bowel movements per day on day four of treatment, so the patient was started on fidaxomicin 200 mg twice a day. After three days of fidaxomicin, his watery bowel movement resolved entirely, and the patient completed ten days of fidaxomicin, and his symptoms completely resolved.

Case 5

A 54-year-old female with a past medical history of end-stage renal disease (ESRD) on hemodialysis, human immunodeficiency virus (HIV) on Biktarvy, hypertension (HTN), and chronic obstructive pulmonary disease (COPD) presented to the ED complaining of abdominal pain and persistent bouts of watery diarrhea approximately two weeks after completing a course of Augmentin and azithromycin for presumed community-acquired pneumonia. About two weeks after completing the antibiotics, she began to experience severe abdominal cramps with persistent watery diarrhea of eight to 10 episodes a day. She denied nausea, vomiting, or fever. A physical exam revealed a blood pressure of 100/58 mmHg, a pulse rate of 95 BPM, and afebrile. An abdominal exam was significant for hyperactive bowel sounds with tenderness over the left lower abdominal quadrant. CBC showed leukocytosis with WBC of 15.9x10^3 cells/μL (normal range 4.0-10.0x10^3 cells/μL) with a neutrophil predominance of 78.9%. CRP was 5.5 mg/dL (normal range 0.00-0.8 mg/dL). Albumin was 3.1 g/dL (normal range: 3.2-4.8 g/dL).

An abdominal CT scan with oral contrast revealed thickened transverse and descending colonic walls with mesenteric fat stranding suspicious for acute inflammatory colitis. The stool exam for ova and parasites, as well as the enteric pathogen panel for Salmonella and Shigella, was negative. *C. difficile *PCR and the toxin test were positive. The patient was started on oral vancomycin 125 mg every six hours. After three days of therapy, the patient continued to have abdominal cramps with several bouts of watery diarrhea of at least eight a day. WBC downtrended to 13.2x10^3 cells/μL (normal range 4.0-10.0x10^3 cells/μL), with CRP downtrending to 3.5 mg/dl (normal range 0.00-0.8 mg/dl). The patient was switched to oral fidaxomicin 200 mg twice a day due to suboptimal clinical response to the vancomycin on the third day. After 48 hours on fidaxomicin, she noted significant improvement in abdominal cramps; there was a reduction in her bowel movement to three to four loose bowel movements/day. Her WBC was down to 9.3x10^3 cells/μL (normal range 4.0-10.0x10^3 cells/μL). After completion of the 10-day fidaxomicin regimen, interval follow-up showed complete resolution of her symptoms.

## Discussion

As a spore-forming bacteria, *C. difficile* is frequently encountered in the environment, especially in medical settings. The prevalence of CDI has been rising worldwide, which is a problem for healthcare systems [7]. The main method of infection is nosocomial transmission, which puts those who have used antibiotics at higher risk [8]. Region-specific and temporal variations in the incidence of *C. difficile* are caused by various variables, including antibiotic usage, infection control measures, and the introduction of novel strains [9]. Although the proportion of people affected may differ, estimates indicate that *C. difficile* is a major contributor to healthcare-associated illnesses worldwide. Gender is not a significant factor in the prevalence of *C. difficile* infections [10]. Widespread *C. difficile* is present in various settings, such as soil, water, and some animals' intestines [11]. However, almost all of the published research has been on CDI that is identified and managed in an acute care hospital context; it does not assess the burden on patients who are not hospitalized, such as those in long-term care institutions, outpatients, and recently released patients. Improved surveillance techniques are required to track the incidence, pinpoint at-risk groups, and describe the genetic epidemiology of the strains causing CDI.

The oral-fecal pathway is the route of transmission. Spores are latent cells extremely resilient to environmental factors, such as antimicrobials and certain disinfectants, which typically target metabolically active cells [12]. Since vegetative (metabolically active) cells of obligate anaerobic bacteria are unlikely to survive the acidic stomach environment or the oxygenated environment outside the host, spores are considered the infectious vehicle [13]. The host microbiota and its metabolome significantly impact the spores' tendency to overrun and colonize the intestine. For instance, changes in the microbiota brought on by antibiotics may create an environment favorable to CDI [14]. Mucolytic enzymes and toxins the bacteria release, including the cell surface protein Cwp84, break the colonic mucosa down [15]. The adherence of bacteria to colon epithelial cells has been demonstrated to be influenced by bacterial cell surface-associated proteins in vitro [16]; alterations in the genes encoding these proteins, or in the genes encoding proteins involved in their processing, typically reduce pathogenicity. The drugs ampicillin and clindamycin increase the expression of at least a subset of colonization factors by the bacteria, such as cell surface protein Cwp84 and surface layer protein A (SlpA) [17].

Clinical manifestations of CDI include gastrointestinal symptoms, including self-limiting diarrhea to fulminant colitis, toxic megacolon, intestinal perforation, pseudomembranous colitis, and multiple organ failure syndrome [18,19]. The diverse criteria employed in guidelines reflect the wide range of potential severity assessments for CDI, which results from the highly variable characteristics of patients who can develop the disease (e.g., age, medication use, underlying comorbidities). When *C. difficile*-related severe diarrhea occurs, profound ulcerations and hemorrhaging pseudomembranous colitis are frequently seen on endoscopy [20,21]. The exceptional rarity of extra-intestinal CDI manifestations, such as bacteremia, highlights that various CDI signs and symptoms are caused by the localized effects of toxins linked to a depleted intestinal flora. An ultrasensitive cell-based assay can identify *C. difficile* toxins in sera from CDI patients. However, more research is needed to determine whether levels of toxicology and severe CDI are related [22].

The existence of clinical manifestations in conjunction with a laboratory test is the cornerstone for diagnosing CDI. Toxin-detecting tests are specific to CDI, but bacterium-detecting tests may show colonization rather than illness. Overdiagnosis and overtreatment are likely to occur from the exclusive use of molecular testing for CDI diagnosis in the absence of toxicological testing [23]. Spores can remain on surfaces and are immune to many disinfectants and alcohol-based hand washes, and they are highly contagious; rapid diagnostic tests should be carried out if a patient is thought to have CDI so treatment can start as soon as possible. Furthermore, therapy should always be paired with patient isolation as spores carry a significant risk for environmental contamination [12]. The earlier Infectious Disease Society of America (IDSA) guideline from 2010 suggested oral metronidazole as first-line therapy for mild to moderate CDI [20]. However, recent information indicates that oral vancomycin is more beneficial than metronidazole in these patients [24]. In addition, compared to vancomycin, metronidazole use may be linked to increased recurrence rates [21]. Moreover, metronidazole should not be administered to patients with comorbidities, the elderly, or women who are pregnant or nursing. Due to these findings, the 2017 Clinical Practice Guidelines for *Clostridium difficile* infection published by the IDSA and the Society for Healthcare Epidemiology of America eliminated metronidazole as the recommended treatment for first episodes of non-severe CDI [25]. As an acceptable therapeutic option for non-severe CDI, the current IDSA guidelines recommend using either fidaxomicin or vancomycin orally. Fidaxomicin is preferred over vancomycin, with a moderate certainty of evidence supporting its significant amount of beneficial effects and safety. Even among patients with a history of recurrent CDI, the narrow-spectrum antimicrobial fidaxomicin reduces the rate of recurrent CDI to approximately 15-20% [6].

The first macrolide antibiotic medication authorized for the treatment of CDI is fidaxomicin. It prevents the first separation of the bacterial DNA strands. In essence, it prevents RNA synthesis from starting extremely early in that route [26]. In the literature, fidaxomicin was linked to a significantly decreased risk of CDI recurrence compared to vancomycin. However, compared to vancomycin, there was no discernible correlation between the usage of fidaxomicin and the CDI clinical cure rate. Analysis indicates that fidaxomicin has a statistically significant lower recurrence rate and a better prolonged clinical response, which aligns with the newly updated clinical practice guidelines by the IDSA [27,28]. Fidaxomicin alters the intestinal microbiota of *C. difficile*-infected individuals less than vancomycin does, both before and after treatment, which may explain why patients have reduced rates of relapse after treatment [29]. In addition to having a smaller antibacterial spectrum than vancomycin, fidaxomicin also prevents sporulation. According to Louie et al., fecal spore counts in patients treated with fidaxomicin were lower than those in individuals treated with vancomycin between 21 and 28 days after therapy [30]. Fidaxomicin has been demonstrated in numerous studies to exhibit significantly greater in vitro activity against *C. difficile* than vancomycin, along with a longer-lasting post-antibiotic impact [31].

## Conclusions

The narrow-spectrum antibiotic fidaxomicin targets *C. difficile* exclusively. It maintains the healthy gut microbiota while preventing the growth of *C. difficile*. The instances in which *C. difficile* patients responded well to fidaxomicin but were not improved on vancomycin demonstrate how CDI treatment is changing. The clinical importance of fidaxomicin comes from its potential to be a helpful substitute, especially when conventional medicines are not working. Nevertheless, given the limits of the available data, care must be taken when evaluating these results. As the discipline develops, more investigations utilizing larger cohorts and rigorous study designs are necessary to determine the relative effectiveness of vancomycin versus fidaxomicin in treating CDI.

## References

[1] Townsend CM, Beauchamp RD, Evers RM, Mattox KL (2016). Sabiston Textbook of Surgery: The Biological Basis of Modern Surgical Practice. https://books.google.com/books?hl=en&lr=&id=KYstDAAAQBAJ&oi=fnd&pg=PP1&dq=Townsend+Jr,+Courtney+M.+%22JR.,+R.+Daniel+Beauchamp,+B.+Mark+Evers,+Kenneth+L.+Mattox.+Sabiston+Textbook+of+Surgery:+The+Biological+Basis+of+Modern+Surgical+Practice&ots=I9M2jfArKv&sig=ADy7BRJ9fKf903J8YetaOuCKm7I#v=onepage&q=Townsend%20Jr%2C%20Courtney%20M.%20%22JR.%2C%20R.%20Daniel%20Beauchamp%2C%20B.%20Mark%20Evers%2C%20Kenneth%20L.%20Mattox.%20Sabiston%20Textbook%20of%20Surgery%3A%20The%20Biological%20Basis%20of%20Modern%20Surgical%20Practice&f=false.

[2] Vassallo A, Tran MC, Goldstein EJ (2014). Clostridium difficile: improving the prevention paradigm in healthcare settings. Expert Rev Anti Infect Ther.

[3] Feuerstadt P, Boules M, Stong L, Dahdal DN, Sacks NC, Lang K, Nelson WW (2021). Clinical complications in patients with primary and recurrent Clostridioides difficile infection: a real-world data analysis. SAGE Open Med.

[4] De Roo AC, Regenbogen SE (2020). Clostridium difficile Infection: an epidemiology update. Clin Colon Rectal Surg.

[5] Fu Y, Luo Y, Grinspan AM (2021). Epidemiology of community-acquired and recurrent Clostridioides difficile infection. Therap Adv Gastroenterol.

[6] Johnson S, Lavergne V, Skinner AM, Gonzales-Luna AJ, Garey KW, Kelly CP, Wilcox MH (2021). Clinical Practice Guideline by the Infectious Diseases Society of America (IDSA) and Society for Healthcare Epidemiology of America (SHEA): 2021 Focused Update Guidelines on Management of Clostridioides difficile Infection in Adults. Clin Infect Dis.

[7] Finn E, Andersson FL, Madin-Warburton M (2021). Burden of Clostridioides difficile infection (CDI) - a systematic review of the epidemiology of primary and recurrent CDI. BMC Infect Dis.

[8] Dubberke E (2012). Strategies for prevention of Clostridium difficile infection. J Hosp Med.

[9] Ho J, Wong SH, Doddangoudar VC, Boost MV, Tse G, Ip M (2020). Regional differences in temporal incidence of Clostridium difficile infection: a systematic review and meta-analysis. Am J Infect Control.

[10] Abukhalil AD, AbuKhdeir L, Hamed M, Al Shami N, Naseef HA, Aiesh BM, Sabateen A (2021). Characteristics, risk factors, and prevalence of Clostridioides difficile among hospitalized patients in a tertiary care hospital in Palestine. Infect Drug Resist.

[11] Curry S (2010). Clostridium difficile. Clin Lab Med.

[12] Paredes-Sabja D, Shen A, Sorg JA (2014). Clostridium difficile spore biology: sporulation, germination, and spore structural proteins. Trends Microbiol.

[13] Deakin LJ, Clare S, Fagan RP (2012). The Clostridium difficile spo0A gene is a persistence and transmission factor. Infect Immun.

[14] Buffie CG, Bucci V, Stein RR (2015). Precision microbiome reconstitution restores bile acid mediated resistance to Clostridium difficile. Nature.

[15] Janoir C, Péchiné S, Grosdidier C, Collignon A (2007). Cwp84, a surface-associated protein of Clostridium difficile, is a cysteine protease with degrading activity on extracellular matrix proteins. J Bacteriol.

[16] Lin YP, Kuo CJ, Koleci X, McDonough SP, Chang YF (2011). Manganese binds to Clostridium difficile Fbp68 and is essential for fibronectin binding. J Biol Chem.

[17] Denève C, Deloménie C, Barc MC, Collignon A, Janoir C (2008). Antibiotics involved in Clostridium difficile-associated disease increase colonization factor gene expression. J Med Microbiol.

[18] Dicks LM, Mikkelsen LS, Brandsborg E, Marcotte H (2019). Clostridium difficile, the difficult “Kloster” fuelled by antibiotics. Curr Microbiol.

[19] Sayedy L, Kothari D, Richards RJ (2010). Toxic megacolon associated Clostridium difficile colitis. World J Gastrointest Endosc.

[20] Cohen SH, Gerding DN, Johnson S (2010). Clinical practice guidelines for Clostridium difficile infection in adults: 2010 update by the Society for Healthcare Epidemiology of America (SHEA) and the Infectious Diseases Society of America (IDSA). Infect Control Hosp Epidemiol.

[21] Debast SB, Bauer MP, Kuijper EJ (2014). European Society of Clinical Microbiology and Infectious Diseases: update of the treatment guidance document for Clostridium difficile infection. Clin Microbiol Infect.

[22] Yu H, Chen K, Wu J (2015). Identification of toxemia in patients with Clostridium difficile infection. PLoS One.

[23] Polage CR, Gyorke CE, Kennedy MA (2015). Overdiagnosis of Clostridium difficile Infection in the molecular test era. JAMA Intern Med.

[24] Song JH, Kim YS (2019). Recurrent Clostridium difficile infection: risk factors, treatment, and prevention. Gut Liver.

[25] McDonald LC, Gerding DN, Johnson S (2018). Clinical Practice Guidelines for Clostridium difficile Infection in Adults and Children: 2017 Update by the Infectious Diseases Society of America (IDSA) and Society for Healthcare Epidemiology of America (SHEA). Clin Infect Dis.

[26] Finegold SM, Molitoris D, Vaisanen ML, Song Y, Liu C, Bolaños M (2004). In vitro activities of OPT-80 and comparator drugs against intestinal bacteria. Antimicrob Agents Chemother.

[27] Al Momani LA, Abughanimeh O, Boonpheng B, Gabriel JG, Young M (2018). Fidaxomicin vs vancomycin for the treatment of a first episode of Clostridium difficile infection: a meta-analysis and systematic review. Cureus.

[28] Tashiro S, Mihara T, Sasaki M (2022). Oral fidaxomicin versus vancomycin for the treatment of Clostridioides difficile infection: a systematic review and meta-analysis of randomized controlled trials. J Infect Chemother.

[29] Louie TJ, Cannon K, Byrne B, Emery J, Ward L, Eyben M, Krulicki W (2012). Fidaxomicin preserves the intestinal microbiome during and after treatment of Clostridium difficile infection (CDI) and reduces both toxin reexpression and recurrence of CDI. Clin Infect Dis.

[30] Louie T, Miller M, Donskey C, Mullane K, Goldstein EJ (2009). Clinical outcomes, safety, and pharmacokinetics of OPT-80 in a phase 2 trial with patients with Clostridium difficile infection. Antimicrob Agents Chemother.

[31] Babakhani F, Seddon J, Robert N, Shue YK, Sears P (2010). Effects of inoculum, pH, and cations on the in vitro activity of fidaxomicin (OPT-80, PAR-101) against Clostridium difficile. Antimicrob Agents Chemother.

